# Large Language Models for Adverse Drug Events: A Clinical Perspective

**DOI:** 10.3390/jcm14155490

**Published:** 2025-08-04

**Authors:** Md Muntasir Zitu, Dwight Owen, Ashish Manne, Ping Wei, Lang Li

**Affiliations:** 1 Department of Machine Learning, Moffitt Cancer Center and Research Institute, Tampa, FL 33612, USA; mdmuntasir.zitu@moffitt.org; 2 Department of Medicine, College of Medicine, The Ohio State University, Columbus, OH 43210, USA; dwight.owen@osumc.edu (D.O.); ashish.manne@osumc.edu (A.M.); 3 Department of Biomedical Informatics, College of Medicine, The Ohio State University, Columbus, OH 43210, USA; ping.wei2@osumc.edu

**Keywords:** large language model, medicine, machine learning, natural language processing, electronic health records, clinical texts, adverse drug events, artificial intelligence

## Abstract

Adverse drug events (ADEs) significantly impact patient safety and health outcomes. Manual ADE detection from clinical narratives is time-consuming, labor-intensive, and costly. Recent advancements in large language models (LLMs), including transformer-based architectures such as Bidirectional Encoder Representations from Transformers (BERT) and Generative Pretrained Transformer (GPT) series, offer promising methods for automating ADE extraction from clinical data. These models have been applied to various aspects of pharmacovigilance and clinical decision support, demonstrating potential in extracting ADE-related information from real-world clinical data. Additionally, chatbot-assisted systems have been explored as tools in clinical management, aiding in medication adherence, patient engagement, and symptom monitoring. This narrative review synthesizes the current state of LLMs in ADE detection from a clinical perspective, organizing studies into categories such as human-facing decision support tools, immune-related ADE detection, cancer-related and non-cancer-related ADE surveillance, and personalized decision support systems. In total, 39 articles were included in this review. Across domains, LLM-driven methods have demonstrated promising performances, often outperforming traditional approaches. However, critical limitations persist, such as domain-specific variability in model performance, interpretability challenges, data quality and privacy concerns, and infrastructure requirements. By addressing these challenges, LLM-based ADE detection could enhance pharmacovigilance practices, improve patient safety outcomes, and optimize clinical workflows.

## 1. Introduction

Adverse drug events (ADEs) are injuries or harms experienced by patients due to medication use [1,2,3]. These events are a major source of patient morbidity and hospital admissions [4]. Monitoring, early detection, and management of ADEs are critical for pharmacovigilance and patient safety [5]; however, much of the relevant information is buried in unstructured forms of texts such as clinical notes. Natural language processing (NLP), the process of automatically analyzing text, has emerged as a tool to extract ADE information from unstructured records.

Traditionally, healthcare institutions have relied on manual chart review to detect ADEs. Indeed, expert review of patient records has been considered the “gold standard” for ADE identification, but it is highly labor-intensive and expensive [5,6]. For example, one study noted that manual review of discharge summaries and clinic notes consumed a major portion of investigator time [5,6]. To support manual review, researchers developed rule-based algorithms. More recent efforts employ machine-learning and deep-learning-based classifiers. These approaches flag potential ADEs by using predefined keywords, changes in lab values, patterns in patient data, or learned associations within the data itself [2,5,6]. Such approaches can be used to scan large datasets more quickly than humans can, but they often miss nuanced or novel cases.

Recent advances in NLP have leveraged large language models (LLMs) to address this problem [3,4]. LLMs are advanced automated systems trained on vast amounts of text to understand and generate language in a way that mimics how humans use language, enabling them to perform a wide range of language-related tasks [7]. Examples include bidirectional encoder representations from transformers (BERT) and the Generative Pretrained Transformer (GPT) series [8]. These models can capture context and semantics in clinical text far beyond simple pattern matching. Importantly, LLMs can be fine-tuned on medical corpora (e.g., ClinicalBERT, BioBERT) or used in few-shot prompting to recognize medical entities. Because ADEs are often only briefly mentioned in clinical notes [9] that contain significant amounts of information not relevant to the ADE, LLMs’ ability to interpret raw text makes them well-suited to extract ADE mentions and drug-event relations that previous methods would miss.

**Figure 1:**
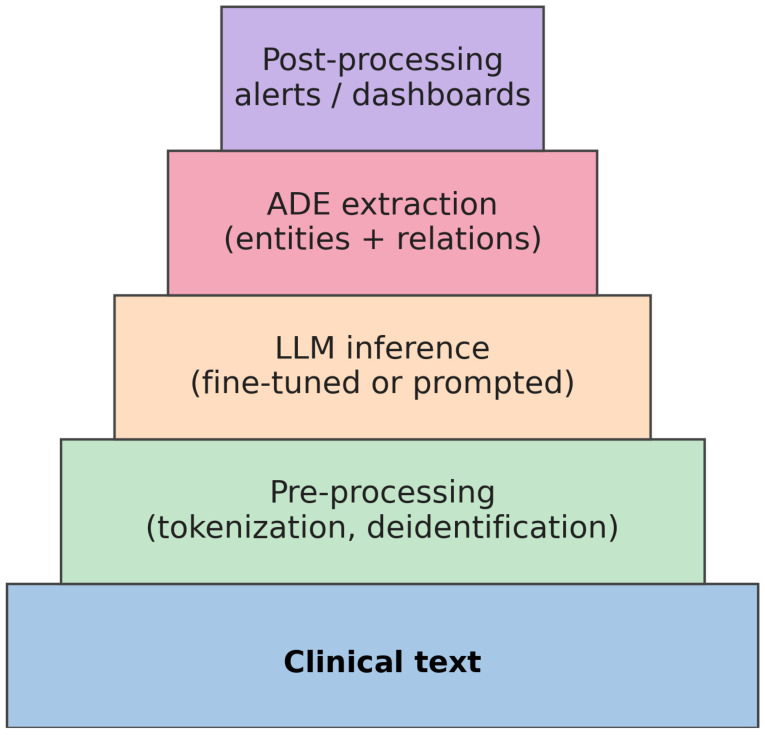
LLM-based ADE extraction workflow, transforming from raw clinical texts through pre-processing, model inference, and ADE extraction to focused post-processing outputs.

The clinical implications of LLM-based ADE detection are substantial. By automating the analysis of narrative notes, these models have the potential to accelerate pharmacovigilance and support decision-making. Studies suggest that LLMs can improve early identification and classification of ADEs and even assist with medication management decisions [10]. Transformer-based models have demonstrated promising accuracy in recognizing drug and event entities [10], which could facilitate more timely and precise ADE monitoring. Integrating LLM output into clinical workflows might help clinicians prevent harm by alerting them to emerging safety signals or prescribing risks. A typical ADE-LLM pipeline, from raw clinical texts to clinician-facing alerts, is outlined schematically in Figure 1.

This review will provide an overview of current clinical use cases of LLMs in ADE detection (e.g., entity and relation extraction from notes), and examine reported performance results using metrics like sensitivity, specificity, F1-score, and AUC from a clinical perspective. We will discuss the benefits these tools may bring, such as scalability and automated surveillance. We will also discuss their limitations, including potential bias, data privacy concerns, and interpretability issues. In doing so, we aim to provide a clear understanding of the promise and challenges of LLM-based ADE detection from a clinical perspective. Please see Table 1 for the Glossary of key terms used in this review.

## 2. Materials and Methods

### 2.1. Purpose and Scope of the Review

Our narrative review aims to synthesize the current state of work on the use of LLMs for ADE detection from a clinical perspective, which includes the clinical impact, advancements, and translational relevance. Unlike systematic reviews that adhere to rigid inclusion criteria and exhaustively capture all available literature, our narrative review adopts a flexible approach [11,12,13,14]. We reviewed articles, including peer-reviewed studies, conference papers, preprints, abstracts, full-text evaluations, and use cases that apply LLMs to clinical data. Our work aims to map the integration of LLMs into pharmacovigilance, retrospective data analysis, clinical decision support, and patient engagement. This includes examining task-specific architectures (e.g., BERT variants), zero- and few-shot prompting, multi-source data fusion, and chatbot-based interventions. We also incorporated editorials and viewpoint articles to reflect expert opinions on the challenges of implementation, such as model generalizability, interpretability, and ethical oversight. This review offers a clinically grounded overview of both the potential and limitations of LLM-based ADE detection systems, identifying key directions for future research and deployment.

### 2.2. Inclusion and Exclusion Criteria

We included studies that focused on LLMs for the detection, classification, or management of ADEs using text-based clinical data. We excluded systematic reviews, meta-analyses, and studies focused solely on non-text-based or non-clinical data, such as omics, molecular, imaging, non-clinical corpora, and social media data.

### 2.3. Literature Search

We conducted a comprehensive literature search across three major databases—PubMed, Embase, and Ovid Medline—targeting publications from 2017 to May 2025. The search was designed to capture studies involving LLMs in the context of clinical ADEs. We set 2017 as the starting point because that was the year the transformer architecture, the foundation of virtually all modern large language models, was introduced [15]. The search strategy incorporated terms spanning three key dimensions: advanced language modeling technologies (e.g., “large language models”, “BERT”, “GPT”, “transformers”, “clinicalBERT”, “PubMedBERT”), adverse event terminology (e.g., “adverse drug events”, “drug toxicity”, “pharmacovigilance”, “medication error”), and data types related to clinical practice (e.g., “clinical notes”, “EHRs”, “unstructured clinical text”). Boolean operators (“AND”, “OR”) were used to combine terms such as ((“large language model” OR BERT OR GPT OR ChatGPT OR transformer …) AND (“adverse drug event” OR “drug toxicity” OR pharmacovigilance OR “medication error” …) AND (“clinical note” OR “electronic health record” OR EHR OR “unstructured clinical text” …)). The searches were restricted to English-language articles appearing in titles or abstracts. After deduplication, a total of 333 distinct articles were initially identified that satisfied the searching criteria.

### 2.4. Article Selection for Review

Following the initial identification of 333 articles, a detailed manual review of titles and abstracts by two independent reviewers was conducted to assess alignment with predefined inclusion criteria—namely, articles that contained clinical data, text-based content, and employed LLM techniques. Articles were excluded if they were non-English, used non-clinical or non-text data, or did not involve LLMs. After applying these criteria, 39 articles were retained for the review.

#### Rationale for Article Categorization

**Figure 2:**
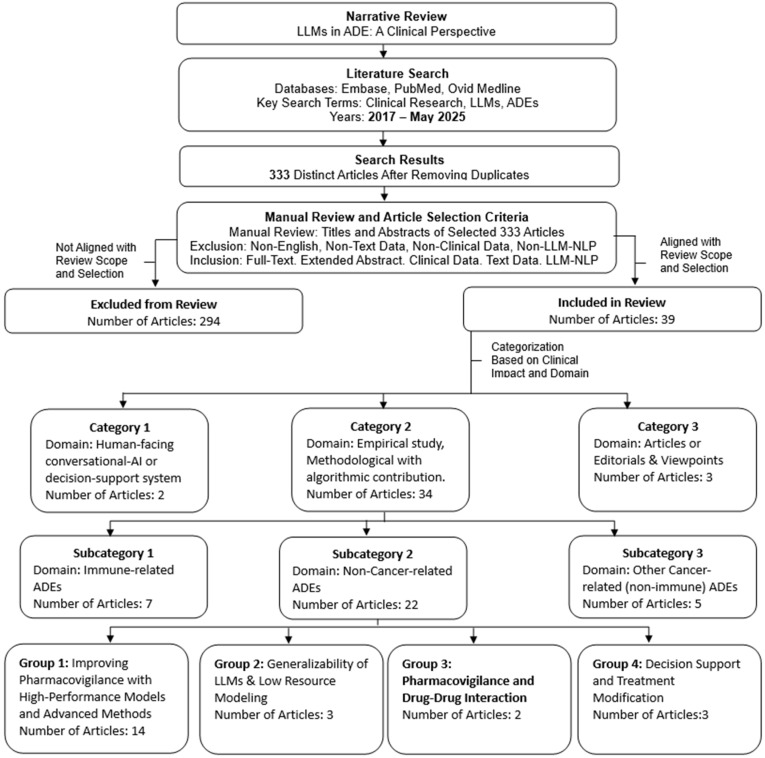
Distribution of the 39 included studies by primary category. Category 2 is further split into three subcategories: immune-related irAEs (*n* = 7), non-cancer ADEs (*n* = 22), and other cancer-related non-immune ADEs (*n* = 5). Only the non-cancer subcategory is further partitioned into groups: high-performance pharmacovigilance (14), generalizability/low-resource (3), drug–drug interactions (2), and decision support/treatment modification (3).

To ensure consistency and avoid placing the same article in multiple categories, we adopted a hierarchical scheme: each study was assigned to one and only one primary category based on its type or dominant objective. Under this rule, the studies fell into three primary categories: 1. human-facing conversational AI or decision support systems (2 articles); 2. empirical studies with algorithmic contributions (34), further divided into subcategories, namely immune-related (7), non-cancer (22), and cancer (non-immune) ADE subcategories; and 3. editorials and viewpoints (*n* = 3). In addition, to address heterogeneity within the non-cancer subcategory articles, we logged recurring thematic groupings as secondary descriptors without fragmenting the main narrative. This recurring thematic grouping includes (a) improving pharmacovigilance with high-performance models and advanced methods (14 articles), (b) generalizability of LLMs and low-resource modeling (3), (c) pharmacovigilance and drug–drug interaction (2), and (d) decision support and treatment modification (3). These themes highlight methodological advances and clinical challenges and may therefore recur across multiple primary categories, subcategories, or groups, even though each article is counted only once in the hierarchy. This dual-level approach preserves mutually exclusive article assignments while acknowledging thematic overlaps, facilitating a structured analysis of the literature. Figure 2 illustrates the entire process in detail.

## 3. Results

Consistent with the hierarchical scheme outlined in Section 2.4, the results of this review are organized according to the categories, subcategories, and groups (only applied to non-cancer subcategory) illustrated in Figure 2. Each table corresponds to a specific domain of work identified through our analysis. Table 2 presents studies under Category 1 (Human-facing conversational AI or decision support system). Under Category 2 (Empirical studies with algorithmic contributions), we group studies into three subcategories: Subcategory 1 includes the immune-related adverse events studies summarized in Table 3, while Subcategory 3 includes the cancer-related but non-immune ADE studies shown in Table 4. Subcategory 2, covering non-cancer-related ADEs, is further divided into groups: generalizability and low-resource modeling (Table 5), high-performance pharmacovigilance methods (Table 6), and drug–drug interaction-focused studies (Table 7). Additional studies addressing treatment modification and personalized decision support are summarized in Table 8. Lastly, Category 3, which includes perspectives and viewpoints on AI in pharmacovigilance, is captured in Table 9.

## 4. Discussion

### 4.1. Human-Facing Conversational AI or Decision Support System

**Figure 3:**
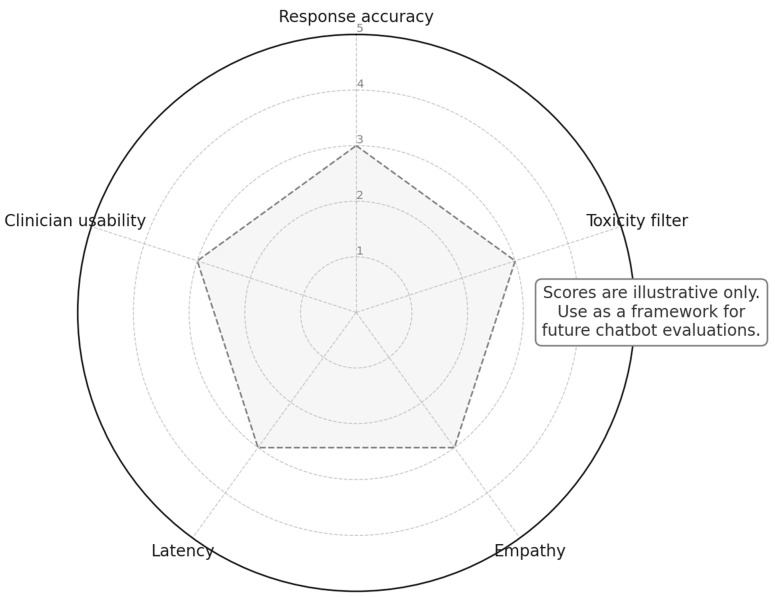
Conceptual evaluation framework for patient-facing ADE chatbots. The diagram is just an illustration and can be used as a template for comparative studies.

LLM-based conversational AI and decision support systems illustrated promising aspects in two complementary ways in delivering actionable ADE information directly to clinicians, significantly advancing medication safety practices: (i) real-time conversational agents that provide point-of-care safety advice Sridharan et al. [16] and (ii) interactive research dashboards that help clinicians visualize and analyze ADE patterns in large datasets (Lenehan et al. [17]). Sridharan et al. [16] evaluated GPT-4’s capability to detect potential drug–drug interactions (DDIs) and dosing errors from clinical scenarios involving 20 commonly prescribed drugs. The authors report that GPT-4 identified DDIs with promising accuracy and provided dosing guidelines; however, they noted occasional critical oversights in interactions, indicating a persistent need for clinical supervision. In a separate complementary study, Lenehan et al. [17] introduced “Clinical nSights,” a user-friendly platform that combines BERT-based NLP models, HIPAA-compliant de-identification algorithms, and knowledge-graph data harmonization to facilitate robust oncology analyses from unstructured clinical records. The authors demonstrate that their platform enables clinicians to intuitively construct patient cohorts based on complex criteria, such as tumor biomarker status (e.g., HER2), immune-related checkpoint-inhibitor toxicities, and detailed symptomatology, without requiring coding expertise. They emphasize Clinical nSights’ capacity to quickly visualize outcomes, such as overall survival and hospitalization rates, significantly streamlining real-world data analyses previously hindered by labor-intensive data curation processes. These systems demonstrated the potential of a meaningful leap forward in clinical informatics, facilitating clinician-friendly ADE assessments while highlighting the ongoing importance of maintaining human supervision in interpreting AI-generated insights. Figure 3 illustrates a five-axis framework as an example that can be used to evaluate ADE chatbots on accuracy, toxicity filtering, empathy, latency, and clinician usability. To make its intended use concrete, consider a routine scenario, such as Simvastatin-associated myalgia: A 55-year-old male presents to the portal with new diffuse muscle pain while taking simvastatin. A clinician-ready chatbot should be capable of (a) *Accuracy*: correctly extracting drug, symptom, temporality/severity and linking myalgia to statin exposure; (b) *Toxicity filtering*: revealing red-flag features (such as weakness and dark urine) and advising urgent care only when indicated; (c) *Empathy*: acknowledging symptoms and uncertainty in supportive language; (d) *Latency*: returning guidance within a few seconds so triage is not delayed; and (e) *Clinician usability*: generating a structured hand-off (problem list, confidence, suggested labs such as creatine kinase (CK), and a concise note snippet) that a clinician can accept/modify.

#### Communicating Outputs to End-Users

For clinicians, outputs should arrive as a structured alert containing (i) the extracted entities and relation (drug, event, timing, severity); (ii) a short rationale/evidence snippet (highlighted tokens or sentences); (iii) a confidence score with simple calibration bands (e.g., high/medium/low); and (iv) a recommended next action (e.g., “order CK and review statin need,” or “escalate high-risk DDI to pharmacy”). For patients, messages should use plain language with readability targets, empathetic phrasing, and safety-tier triage (self-care vs. contact clinic vs. urgent care/ED). For research/regulatory users, dashboards should expose aggregated rates with uncertainty (CIs), calibration plots, subgroup fairness slices, and a complete audit trail (model version, prompts, thresholds, and overrides). These communication elements complement our five-axis framework (accuracy, toxicity filtering, empathy, latency, and clinician usability) and the interpretability example in Figure 3.

### 4.2. Empirical Studies with Clinical Relevance

#### 4.2.1. Immune-Related ADEs

IrAEs constitute a distinct and clinically impactful subset of ADEs where NLP could play a critical role in reducing manual review efforts as well as improving clinical outcomes [55,56]. Recent studies demonstrate that LLM-driven approaches can play a critical role in improving the detection and management of immunotherapy-related adverse events (irAEs), enabling earlier intervention and reducing clinician workload. For example, Bejan et al. [19] introduced the multi-institutional “irAE-GPT” system, leveraging a zero-shot GPT-4o model. This approach achieved moderate accuracy across three different clinical cohorts (Vanderbilt, Roche, and UCSF), with micro-F1 scores ranging from 0.56 to 0.66. Notably, the authors reported high sensitivities (range: 0.74–0.88) and specificities (range: 0.85–0.94), but moderate positive predictive values (PPVs), indicating a clinical tendency toward overpredicting irAEs.

Block et al. [21] fine-tuned SciBERT models specifically on oncology electronic health record (EHR) notes to extract irAEs, reporting a high F1 performance of approximately 0.85. This NLP system highlighted improvement in workflow efficiency, reducing the manual chart review time from nine weeks to merely ten minutes in a cohort of approximately 9000 patients.

In a different study, Barman et al. [18] utilized a fine-tuned SciBERT model, achieving a patient-level F1 score of 0.84. This NLP approach notably identified clinically significant adverse events such as myocarditis, encephalitis, pneumonitis, and severe cutaneous adverse reactions, which traditional ICD-based coding frequently missed. Clinically, NLP identified 30% more cases than diagnosis codes alone, resulting in clinically actionable outcomes such as corticosteroid initiation in 82% of cases and permanent immune checkpoint inhibitor (ICI) discontinuation in 35.9% of cases.

Sun et al. [24] conducted a hospital cohort study explicitly comparing an LLM pipeline to conventional ICD-based diagnostic codes, reporting significantly improved sensitivity (94.7%) for severe irAE detection at the patient level. This NLP-driven approach showed the potential to accelerate clinical adjudication processes, reducing time per case from approximately 15 min to just 9.5 s—a clinically meaningful 160-fold efficiency gain. In another targeted investigation, Sun et al. [23] developed a specialized LLM-based screening tool for myocarditis, achieving exceptional sensitivity (95.5%) and specificity (95.4%) within one day of hospital admission.

Guillot et al. [20] explored the use of GPT-4 in extracting CAR T-cell therapy-related adverse events from clinical notes, achieving a moderate accuracy of approximately 64% at the note-level in a zero-shot setting. Despite moderate accuracy, the automated NLP-driven extraction notably reduced clinician review burdens, which was particularly impactful given the severity and complexity of CAR T-related adverse events, such as hyponatremia, leukocytosis, and encephalopathy. This underscores the potential clinical utility of GPT-based systems even in preliminary, zero-shot applications.

Petersen et al. [22] employed an AI-driven language model in a real-world cohort of melanoma patients experiencing immunotherapy-related colitis. They reported that biologic therapy was clinically necessary in 45% of patients, uncovering significant clinical implications regarding glucocorticoid use exceeding clinical guidelines. Importantly, biologic treatment initiation was frequently delayed, highlighting the necessity for improved clinical decision support tools. These insights emphasize the potential of AI-informed clinical strategies to optimize IR colitis management, reduce unnecessary prolonged glucocorticoid use, and expedite biologic therapy initiation.

Collectively, these studies underscore clinical advancements and the growing importance of LLM-driven approaches in managing immunotherapy-related adverse events. They demonstrate the potential for robust clinical utility in terms of early irAE detection, improved efficiency in clinical workflows, accurate pharmacovigilance, and critical support for clinical decision-making. However, they also highlight the ongoing need for improved PPVs and careful consideration of overprediction risks. Additionally, these studies utilized highly selected retrospective cohorts, and to our knowledge, the performance of any of these tools in a prospective trial has not been demonstrated.

#### 4.2.2. Other Cancer-Related (Non-Immune) ADEs

Several studies have tackled chemotherapy- and radiotherapy-associated toxicities with domain-adapted LLMs. Chen et al. [25] fine-tuned PubMedBERT to identify and grade radiation-induced esophagitis from thoracic oncology notes, achieving macro-F1 up to 0.92 and capturing every true case that ICD-10 coding missed. Andrade et al. [26] demonstrated that a Japanese BERT model, further adapted on 800 in-domain documents, uncovered previously unreported chemotherapy ADEs in breast-cancer notes with a micro-F1 of 0.84. Extending large-scale surveillance, Kawazoe et al. [27] mined 8 million multiclass clinical notes to reproduce known hazard ratios for mucositis, thrombocytopenia, and alopecia, illustrating postmarketing signal detection at population scale. Nishiyama et al. [28] combined BERT NER with Levenshtein normalization to flag taxane-induced neuropathy within 30 days (sensitivity ≈ 61%), offering near-real-time symptom tracking. Finally, Yanagisawa et al. [29] applied MedNER to community-pharmacy assessment notes, reaching macro-F1 ≈ 0.72 and enabling pharmacists to monitor outpatient chemotherapy ADEs systematically. Collectively, these LLM-driven approaches broaden pharmacovigilance beyond immunotherapy, covering radiotherapy and cytotoxic regimens across inpatient and community settings.

#### 4.2.3. Non-Cancer-Related ADEs

##### Generalizability of LLMs and Low-Resource Modeling

Recent studies have underscored the importance of robust data resources and efficient pipelines to enhance medication safety surveillance. Dai et al. [30] introduce MultiADE, a multi-domain ADE extraction benchmark spanning electronic health records, medical literature, and social media, including a newly expanded CADECv2 corpus. The authors report that even a state-of-the-art model (GPT-4, five-shot prompting) achieved high micro-F1 for medication entity recognition (~92%) but substantially lower F1 for ADE mentions (≈52–69%) across corpora, underscoring persistent domain-specific performance gaps. By making these heterogeneous datasets publicly available, this work enables unified active ADE surveillance across text types and illuminates the need for improved domain adaptation methods. Similarly, addressing generalizability, Zitu et al. [32] constructed an immune checkpoint inhibitor ADE corpus and evaluated models across institutions. The authors found that a domain-specific transformer (ClinicalBERT) achieved the highest cross-site F1 (~0.74–0.78) when trained on one hospital’s notes and tested on another’s, outperforming conventional classifiers and a generic BERT (F1 ~0.55–0.73). This suggests that domain-tailored language models can better generalize ADE detection across health systems, enhancing system-wide pharmacovigilance. In contrast, Tavabi et al. [31] focused on pragmatic scalability: they developed a low-resource NLP pipeline (SE-K) to extract surgical registry variables from 20 years of pediatric notes, reporting an AUROC of ~0.94 on external validation—outperforming a BERT-based approach (AUROC ~0.87) while running six times faster. The authors emphasize that SE-K’s interpretability and efficiency facilitate rapid registry development for quality improvement and adverse event monitoring. This demonstrates significant translational potential, as faster, resource-light pipelines can accelerate the integration of ADE surveillance into routine clinical workflows.

##### Improving Pharmacovigilance with High-Performance Models and Advanced Methods

Several studies highlighted high performance gains, introducing methodological advancements over previous approaches in their respective tasks relevant to pharmacovigilance. Initial efforts by Li et al. [37] introduced domain-adapted BERT models trained on millions of EHR notes to normalize medications and ADEs, achieving high F1 scores (~90%) on standard benchmarks and demonstrating the feasibility of automated ADE concept extraction in clinical text. Building on this foundation, Mahendran et al. [38] fine-tuned BERT for ADE relation classification on the 2018 n2c2 Track 2 dataset (505 MIMIC-III discharge summaries), achieving precision 0.93, recall 0.96, and lenient micro-F1 0.94, substantially outperforming rule-based baselines and enabling real-time surveillance. Likewise, Chaichulee et al. [33] attained near-human performance (ensemble F1 ≈ 99%) in encoding free-text adverse reaction reports into standardized symptom codes via BERT-based multi-label classification, suggesting that tedious manual coding by pharmacists can be largely automated. Fan et al. [34] focused on dietary supplement (DS) safety, applying a BERT + CRF model for named entity recognition and an attention-based Bi-LSTM for relation extraction; they reported F1-scores of ~0.86 and 0.89 for DS-related adverse events and indications, and noted that this approach enables DS pharmacovigilance to complement spontaneous reports. Hu et al. [35] report that a GPT-4-based few-shot framework can extract complex ADE information from clinical case summaries and vaccine reports with relaxed F1 up to ~0.86, greatly reducing the need for manual annotation. McMaster et al. [39] developed a domain-adapted DeBERTa model for discharge summaries, achieving ROC-AUC 0.955 in flagging documents containing ADR mentions and significantly improving reporting rates by bypassing labor-intensive coding. In parallel, Silverman et al. [40] demonstrated that fine-tuning a specialized “UCSF-BERT” LLM can identify serious treatment-emergent ADEs in outpatient notes (macro F1 ~0.68), surpassing prior NLP models by roughly 5–10% in performance. Similarly, Koon et al. [36] showed that a fine-tuned Bio-ELECTRA transformer improved causal drug–ADE relation classification F1 from 0.64 to 0.74 over a traditional model; the authors added simple clinical rules to further boost precision (~5% gain), highlighting the value of combining domain knowledge with advanced algorithms. 

Guan et al. [41] leveraged a BERT-based contextual model and “near-miss” negative sampling to extract long-range drug–event relations, reporting a 6.4–6.7 point F1 improvement (24–28% error reduction) in ADE and indication relation extraction over previous methods. Mitra et al. [42] evaluated end-to-end bleeding event extraction in EHR notes; their best system (a BiLSTM-CRF) achieved a macro-F ≈0.75 for six bleeding-related entity types, outperforming transformer models on limited data. Notably, when detecting bleeding occurrences at the document level (e.g., identifying patients with any bleed), performance rose to F1 ≈0.96, highlighting that such NLP tools can reliably flag serious ADEs with minimal false omissions. Focusing on relation classification, Rawat et al. [43] showed that fine-tuned transformer models (BioBERT, ClinicalBERT) outperformed CNN and graph-based classifiers by ~1.3–8% absolute F1 (*p* < 0.001), and simple ensembling further boosted overall ADE relation detection accuracy. In parallel, Wu et al. [45] demonstrated that advanced NLP systems can markedly reduce manual review effort: their hybrid BERT–BiLSTM–CRF model achieved 96.2% F1 in recognizing ADE mentions from Chinese reports, substantially outperforming human annotators (~79.5% F1). Likewise, Staneková et al. [44] improved allergy ADE identification by combining synthetic data augmentation with transfer learning to classify penicillin-allergy risk, substantially reducing additional annotation needs; using EHR data from 12,232 patients with 69 synthetic labels and a 1000-case test set, they reported AUC 0.984 (allergy vs. intolerance) and 0.995 (high- vs. low-risk). Wu et al. [46] further illustrated the importance of scalability and adaptability: by using Longformer-based models and optimized note chunking, they achieved robust generalization from a public dataset to hospital notes (macro-F1 ≈0.78 on external data), and highlighted practical considerations like token limits and chunk size for deploying ADE detectors in real-world EHRs. Collectively, these studies demonstrate notable methodological advances—from high-precision entity extraction and relation classification to leveraging LLMs and transfer learning—that translate into clinically relevant improvements in ADE monitoring. The authors also report reduced manual review burden and more timely ADE detection. These innovations can enhance pharmacovigilance and clinical decision support by enabling earlier identification of ADEs and more comprehensive routine safety surveillance. These diverse studies illustrate that modern NLP architectures (from Bi-LSTMs to domain-tuned transformers to GPT-4) have the potential (F1 often 0.7–0.9+, AUC ~0.96) to augment clinical decision support for medication safety, reduce manual review effort, and thus improve pharmacovigilance.

##### Pharmacovigilance and Drug–Drug Interaction

As part of efforts to enhance pharmacovigilance, recent research applied NLP to uncover underrecognized drug–drug interaction (DDI) signals. Jeong et al. [47] introduced a translational pipeline coupling literature-based NLP extraction with real-world data analysis. The authors utilized a fine-tuned SciFive model to extract 160,321 PubMed abstracts for pharmacokinetic DDI–severe adverse drug reaction (SADR) signals, identifying 111 putative interactions. They report that 17 of these were validated via logistic regression in two large EHR cohorts (~3.18 million patients at VUMC and 239,000 participants in All of Us), including 9 novel DDI–SADR associations not documented in DrugBank (Jeong et al. [47]). These newly uncovered interactions, some with high effect sizes (e.g., OR 35.8 for methadone–ciprofloxacin causing respiratory depression), have immediate medication safety implications, enabling clinicians to avoid previously unrecognized high-risk drug combinations. Similarly, Ma et al. [48] leveraged Chinese EMR data and an NLP pipeline (BERT with logistic regression) to identify 20 concomitant medications that elevate the risk of isoniazid-induced liver injury among 3209 treated patients (Ma et al. [48]). The authors highlight cefepime as the strongest risk signal (OR 2.69, *p* = 0.003) for isoniazid hepatotoxicity (Ma et al. [48]). These complementary studies demonstrate how NLP-driven analyses can augment pharmacovigilance by identifying clinically significant DDIs from disparate data sources, ultimately advancing drug safety monitoring and pharmacovigilance.

##### Clinical Decision Support and Treatment Modification

Recent studies have demonstrated the potential of LLM-driven clinical decision support tools, which facilitate more personalized and safer medication management in various domains by informing treatment modifications, discontinuation decisions, and pharmacogenomic dosing. For example, Kumar et al. [49] reports that a fine-tuned transformer QA model achieved 91.4% accuracy in automatically extracting EHR-documented reasons for semaglutide (Ozempic) discontinuation (spanning ~90,000 patients and 1.2 million clinical note sentences), streamlining pharmacovigilance by capturing insights often lost in structured data and aiding clinicians’ discontinuation decision-making. Similarly, Wang et al. [50] attained 94.5% accuracy using a RoBERTa-based QA model (fine-tuned “Gemini Pro 1.5”) to identify seven common reasons for discontinuing or switching biologic therapy in inflammatory bowel disease across four health systems (10.6 million notes), which the authors suggest can inform timely treatment modifications. In the pharmacogenomics domain, Washington et al. [51] applied a GPT-4 prompt-based method to a 100,000-patient EHR–exome dataset for clopidogrel pharmacogenomics. The authors report that CYP2C19 genotype-guided antiplatelet therapy could reduce clopidogrel-related adverse drug events by 38%, preventing roughly one thrombosis per ~30 patients and showcasing a tangible benefit of population-level PGx decision support. These studies collectively illustrate both the clinical impact and methodological versatility of LLM-driven decision support by parsing unstructured clinical narratives at scale (Kumar, Wang) and extracting pharmacogenomic insights via prompt-based LLMs (Washington). Such tools reveal why treatments are stopped or adjusted and help to establish personalized interventions (e.g., genotype-guided dosing). These capabilities have the potential to enhance clinical decision-making at the point of care, improve medication safety, and ultimately optimize patient outcomes.

#### 4.2.4. Articles or Editorials and Viewpoints

Recent reviews and viewpoints highlight the transformative potential of LLMs in enhancing pharmacovigilance practices, with particular emphasis on their strengths and challenges in real-world applications. Matheny et al. [54] highlight the significant opportunities that LLMs offer for postmarketing surveillance, noting their ability to efficiently synthesize information from diverse narrative sources, such as electronic health records (EHRs) and adverse event reports. The authors report that LLMs can effectively identify novel adverse event signals from narrative EHR data and support epidemiologic investigations by probabilistic phenotyping and extracting crucial confounding information. However, they caution about critical challenges, including infrastructure requirements, model drift over time, and risks of generating nonfactual statements (“hallucinations”), stressing the need for meticulous validation processes to avoid misinforming clinical decisions. Similarly, Gallifant et al. [52] emphasize the value of AI and NLP-based tools in real-world oncology pharmacovigilance, proposing a structured “three Ps” framework—processing, pipelines, and patient outcomes—for effective AI integration into clinical workflows. They highlight the complexities involved in attributing adverse events accurately, especially given the variability in documentation and clinician judgment inherent in EHR data. Additionally, they underscore equity considerations, advocating that AI systems must be developed with inclusivity to ensure representative data across diverse patient populations. Lisi et al. [53] expands the discussion by illustrating AI’s broader role, including the utilization of chatbots like ChatGPT for patient education on ADEs, and emphasizes pharmacists’ essential involvement in developing and critically assessing AI-driven pharmacovigilance tools. She discusses significant limitations, including data quality concerns, the accuracy of chatbot-generated medical advice, and the necessity for pharmacists to maintain oversight in the AI-driven medication-use process. Collectively, these articles demonstrate a clear consensus that while LLMs hold promise for advancing pharmacovigilance and improving clinical outcomes, careful attention to methodological rigor, bias mitigation, and ethical oversight is crucial to ensure their safe and effective deployment in clinical practice.

## 5. Limitations

As summarized in Table 10, the well-publicized benefits of LLM-based ADE detection—speed, scalability, and decision support—represent only the visible tip of the iceberg; the larger submerged mass comprises practical hurdles such as domain-specific performance variability, limited interpretability, data-quality and privacy constraints, and the infrastructure required for clinical deployment.

### 5.1. Generalizability

Despite promising recent advancements of LLM-based approaches, ADE detection faces several limitations. Model generalizability across clinical domains remains notably challenging. Studies such as those by Dai et al. [30] and Zitu et al. [32] have demonstrated variability in performance when models trained on specific institutional or textual data sources are tested externally. For instance, Dai et al. [30] specifically reported substantial variability in ADE extraction performance (F1: ~52–69%) across different data sources when shifting domains, indicating potential risks in directly translating these findings into clinical practice without careful validation. Similarly, Nishiyama et al. [28] observed that their approach captured peripheral neuropathy events in breast cancer patients with moderate sensitivity (60.7%) compared to manual review (74%), highlighting notable gaps in temporal coverage and detection completeness. The input data including specific institution as well as patient demographic data may limit generalizability to patients in other institutions or with differing demographic characteristics.

### 5.2. Interpretability

**Figure 4:**
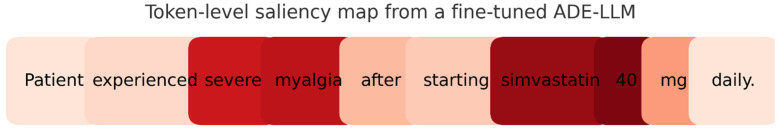
Token-level saliency map from a fine-tuned ADE-LLM. Darker red blocks indicate higher token importance for classifying an adverse drug event. Contiguous sub-word pieces have been merged and displayed as whole words for readability.

Interpretability and clinician trust represent further practical barriers. Sridharan et al. [16] systematically evaluated multiple general-purpose LLMs (ChatGPT, Gemini, Claude, and Llama) with regard to drug–drug interaction (DDI) detection and dosing accuracy, finding that critical interactions (e.g., metoprolol–verapamil) and key dosing guidelines (vancomycin, gentamicin) were frequently missed or incorrectly advised, posing potential patient safety risks. Such model-generated inaccuracies and hallucinations underscore persistent issues with interpretability and reliability. To illustrate what the model “looks at,” Figure 4 shows a token-level saliency map for a note snippet in which the LLM correctly flags myalgia as an ADE associated with simvastatin.

### 5.3. Data Quality

Another explicit challenge is the requirement for extensive annotation and validation efforts. Gallifant et al. [52] noted that poorly defined or inadequately labeled datasets reduce detection accuracy, citing pneumonitis detection rates of 2.9%, which is significantly lower than the clinically expected range of 10–20% when labeling is insufficient. This highlights the importance of rigorous and consistent annotation processes.

Additionally, our review focuses on native EHR narratives; however, important ADE evidence also exists in legacy hard-copy and scanned/handwritten records. These sources introduce OCR and layout/handwriting errors that can degrade extraction. Practical safeguards include document-quality scoring, evidence-linked OCR with page snippets, and route low-confidence or conflicting cases to human review.

### 5.4. Infrastructure

Infrastructure demands and equity considerations pose substantial logistical limitations. Matheny et al. [54] highlighted that LLM-driven pharmacovigilance pipelines require significant computational resources, which complicate equitable access across diverse healthcare systems and potentially exacerbate disparities. Furthermore, Lisi et al. [53] emphasized the pharmacists’ essential role in validating AI-derived ADE recommendations, noting that relying solely on model outputs risks delivering incorrect or unsafe advice without expert oversight.

### 5.5. Regulatory

Additionally, regulatory uncertainty regarding the deployment of LLMs remains an ongoing limitation. Matheny et al. [54] raised critical concerns about the lack of standardized guidelines for evaluating and integrating LLM-based models into FDA Sentinel surveillance processes, particularly regarding the management of false positive signals, hallucinations, and temporal inaccuracies. This regulatory ambiguity poses a substantial hurdle to broader clinical adoption, underscoring the need for transparent validation protocols and explicit regulatory oversight to ensure the reliable and safe implementation of these technologies.

## 6. Future Directions

**Figure 5:**
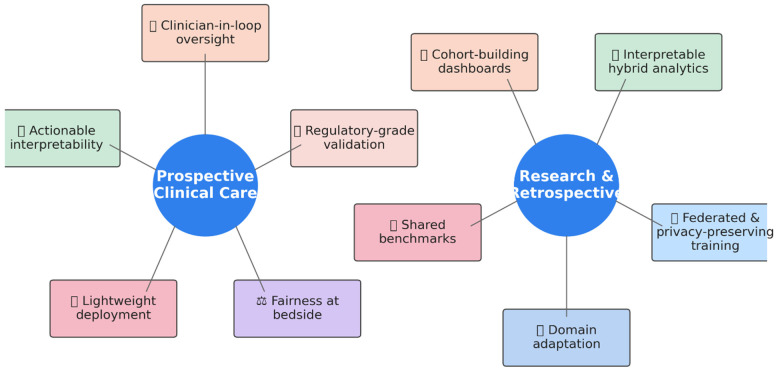
Key future directions for advancing LLM-based ADE detection.

Large-language-model pharmacovigilance is poised to move on two parallel fronts. The first pushes LLMs into prospective clinical care, where the recommendations will be safe, fast, and auditable. The second exploits LLMs in research settings, clinical trial pipelines, and large retrospective datasets, generating evidence at a scale classical NLP never reached. Figure 5 visualizes the twin roadmaps ahead, with (a) prospective clinical deployment on the left and research, and (b) driven development on the right. Each track is surrounded by the key priorities that emerged from this review. Below, a brief narrative introduces the next steps for each track.

### 6.1. LLMs in Prospective Clinical Care

*Vision: real-time, bedside safety intelligence that clinicians can trust.*

Early GPT-4 demonstrations are impressive, yet prospective deployments still falter with regard to context drift, justification, and governance. To cross the last mile, we must improve the human hand-off, make model reasoning visible, and hard-wire equity and regulatory guard-rails into every pipeline.

**Clinician-in-the-loop oversight.** *Always route alerts to a clinician; do not auto-change medications.* For example, a flagged *metoprolol–verapamil* interaction is sent to pharmacy/oncology for confirmation and advice; GPT-4 missed several clinically significant DDIs in simulated scenarios, underscoring a need for pharmacist/physician arbitration before alerts reach the chart (Sridharan et al. [16]).

**Actionable interpretability.** *Show why the alert fired in clinician terms.* For example, highlight the words “simvastatin … myalgia … dark urine” and provide a one-line rationale tied to guidance. Rule-augmented transformers raise precision by ≈5% and highlight causal clues that humans recognize (Rawat et al. [43]; Koon et al. [36]).

**Lightweight deployment.** *Run on standard hospital CPUs (low-resource) when GPUs (resource-heavy) are not available.* For example, SE-K achieved AUROC ≈ 0.94 and ran a lot faster than a BERT baseline on external validation, enabling bedside use in resource-limited settings (Tavabi et al. [31]).

**Fairness at the bedside.** *Check performance against several other factors such as age, sex, race, and site so no group is missed.* For example, build a small dashboard that shows sensitivity/PPV by subgroup. Gallifant et al. [52] warn that under-represented demographics still receive weaker ADE detection; thus, bias audits and balanced pretraining are mandatory.

**Regulatory-grade validation.** *Keep an audit trail and prospectively test before routine use.* FDA-aligned audit trails for LLM phenotypes (Matheny et al. [54]) foreshadow the evidence bar real-time systems must clear before widespread use.

**Reliability and hallucination mitigation.** Pair evidence-linked generation with layered guard-rails to mitigate hallucination and make the output more reliable for the end users. Apply several steps such as (i) retrieval-augmented outputs with cited information source; (ii) safety checks for DDIs, contraindications, and dose/unit/lab limits; (iii) calibrated confidence with an abstention-and-escalation policy for when the confidence is low or when outputs conflicts with existing evidence; (iv) agreement gating using a supervised detector or ensemble before surfacing high-risk alerts; (v) exposing a simple reliability score (high/medium/low) derived from confidence calibration, evidence coverage, and model agreements; (vi) applying low triggers abstention and clinician review; (vii) operations dashboards track PPV, override rate, time to action, calibration error and (viii) logging all inputs, prompts, model versions, thresholds, outputs, and reasons for future reference.

### 6.2. LLMs in Research: Clinical Trials and Retrospective Analyses

*Vision: rapidly scalable evidence generation across vast historical corpora.*

Research pipelines are more tolerant of latency but unforgiving about reproducibility and privacy. The immediate agenda is to stabilize performance across sites, share weights without sharing data, and couple LLM outputs with interpretable causal analytics.

**Domain adaptation and continual learning.** Fine-tuning or synthetic augmentation boosts F1 by 6–10% as vocabularies drift (Guan et al. [41]; Staneková et al. [44]).

**Federated, privacy-preserving training.** Multi-institution modeling—without centralizing raw notes—is essential for multi-site trials and cross-market surveillance.

**Interpretable hybrid analytics.** Transformer + log-reg ensembles provide audit-friendly causality scores suitable for regulatory dossiers (Rawat et al. [43]).

**Cohort-building dashboards.** Clinical nSights condenses ADE cohort assembly from weeks to minutes, accelerating protocol design for immune-toxicity or oncology trials.

**Shared benchmarks.** Public tasks such as MADE and n2c2 remain the sandbox for stress-testing new methods before prospective roll-out.

**Inclusion of legacy documents.** Research pipelines should integrate OCR/handwriting recognition with reliability scoring and abstention, so legacy documents feed safely into the same LLM framework.

Pursuing these twin tracks—bedside integration and large-scale research—will turn LLM pharmacovigilance from promising prototypes into trustworthy, regulation-ready tools that measurably reduce the burden of adverse drug events. Table 11 provides a brief summary of future directions in both the near and longer terms.

## 7. Conclusions

Recent advancements in utilizing LLMs have the potential to enhance capabilities in detecting and managing ADEs. Studies consistently report improved accuracy and efficiency when applying LLMs to real-world clinical data.

However, substantial barriers remain, notably in terms of domain generalizability, interpretability, and reliability.

Future research should prioritize systematic domain adaptation methods such as intermediate transfer learning, synthetic data augmentation, and hybrid modeling approaches to enhance both generalizability and interpretability. Overall, promising clinical advancements have been made in applying LLMs for ADE detection. Addressing the remaining methodological, practical, and regulatory challenges will be vital for ensuring the reliable, equitable, and effective integration of LLM-based ADE detection systems into clinical and pharmacovigilance workflows.

## Figures and Tables

**Table 1:** Glossary of key terms.

Term	Definition
Accuracy	The proportion of correctly predicted instances among all instances evaluated (overall correctness).
ACH	Adrenal insufficiency, colitis, hypophysitis; shorthand for three common immune-related adverse events involving adrenal glands, the colon, and the pituitary gland (seen in immunotherapy).
ADE	Adverse Drug Event—any harmful or unintended event caused by the use of a medication.
AI	Artificial Intelligence—the simulation of human cognitive processes by machines or computer systems.
AllergyRoBERTa	A domain-adapted RoBERTa-based model designed for classifying allergy-related information in EHRs (electronic health records).
AUROC	Area Under the Receiver Operating Characteristic (ROC) Curve—a metric for evaluating how well a classifier separates classes (also known as AUC).
BERT	Bidirectional encoder representations from transformers—a transformer-based language model pretrained on large text corpora, used as a foundation for many NLP tasks.
BioBERT	A biomedical domain-adapted version of BERT, pretrained on biomedical literature (PubMed and PMC texts) for biomedical NLP tasks.
ChatGPT	OpenAI’s conversational LLM (part of the GPT series) used in zero-shot and few-shot clinical NLP tasks.
ClinicalBERT	A BERT model fine-tuned on MIMIC clinical notes for healthcare-specific language understanding.
ClinicalLongformer	An adaptation of the Longformer model for handling long clinical documents in ADE extraction tasks (utilizes sparse attention for long contexts).
DDI	Drug–drug interaction—an interaction in which one drug affects the activity or effects of another drug, potentially leading to ADEs.
DeBERTa	Decoding-enhanced BERT with Disentangled Attention—an advanced transformer model (BERT variant) with improved attention mechanisms for NLP tasks.
EHR	Electronic Health Record—a digital version of a patient’s medical chart or history, including diagnoses, medications, and clinical notes.
Entity extraction	The process of identifying and extracting named entities (e.g., medications, symptoms) from unstructured clinical text.
F1 Macro (Macro-F1)	The F1-score averaged across all classes, giving equal weight to each class regardless of its frequency (macro-averaged F1).
F1 Micro (Micro-F1)	The F1-score calculated globally across all instances by pooling predictions from all classes (micro-averaged F1).
F1 score	The harmonic mean of precision and recall—a single metric that balances both for classification performance.
Few-shot prompting	A technique where only a few example prompts are provided to an LLM to demonstrate a task, enabling the model to perform the task without additional training.
Fine-tuning	The process of training a pretrained model on a specific new dataset to specialize it for a particular task.
Gemini Pro	An advanced transformer-based large language model tested for performance on clinical NLP tasks.
GPT-4	The fourth-generation model in OpenAI’s Generative Pretrained Transformer series—a state-of-the-art LLM used for complex language tasks, including medical NLP.
HIPAA	Health Insurance Portability and Accountability Act—a U.S. law that mandates the protection and confidential handling of patient health information.
ICD	International Classification of Diseases—a standardized system of codes for diagnoses and health conditions, used worldwide in healthcare documentation.
ICI	Immune Checkpoint Inhibitor—a type of cancer immunotherapy drug (e.g., anti-PD-1 or anti-CTLA-4 antibodies) that unleashes the immune system by blocking inhibitory receptors; often associated with immune-related adverse events.
irAE	Immune-related Adverse Event—an adverse effect of immunotherapy (such as checkpoint inhibitors) where the immune system attacks normal organs (e.g., colitis or pneumonitis).
LLM	Large Language Model—an AI model trained on massive text datasets capable of understanding, generating, and summarizing human-like language.
LLaMA	Large Language Model Meta AI—an open-source family of LLMs developed by Meta (Facebook).
Longformer	A transformer model variant optimized for long documents via sparse attention, allowing it to efficiently process long clinical texts.
MADE	Medication and Adverse Drug Events—a benchmark dataset for evaluating models on ADE extraction tasks.
MEPS	Myocarditis, encephalitis, pneumonitis, and severe cutaneous adverse reactions—an acronym grouping several serious immune-related adverse events often observed during immunotherapy.
MeDeBERTa	A medical-domain variant of DeBERTa fine-tuned for adverse event (ADE) extraction tasks.
MedNERCRJA	A BERT-based Japanese-language medical named the entity recognition model (tailored for Japanese clinical text).
MedNERN	Medical Named Entity Recognition—the task of identifying and classifying medical terms (like drugs, diseases, symptoms) in text.
NER (Named Entity Recognition)	An NLP task of detecting and labeling spans of text that constitute named entities (such as medication names or disease names).
NLP	Natural Language Processing—a field of AI focused on enabling computers to understand and manipulate human language text and speech.
PGx	Pharmacogenomics—the study of how an individual’s genetic makeup affects their response to drugs.
Pharmacovigilance	The monitoring, detection, assessment, and prevention of adverse effects or any drug-related problems, especially after a drug has been released to the market.
Precision	The proportion of retrieved instances that are relevant—in classification, the fraction of positive predictions that are actually correct (true positives divided by total predicted positives).
RAG	Retrieval-Augmented Generation—an approach where an LLM incorporates information retrieved from external sources into its generated responses.
Recall	The proportion of actual positive instances that are correctly identified—also known as sensitivity or true positive rate (true positives divided by total actual positives).
Relation extraction	The task of identifying and classifying relationships between entities in text (for example, determining if a drug is associated with a specific adverse event).
RoBERTa	A Robustly Optimized BERT approach—a transformer model that improves on BERT’s pretraining methodology, leading to better performance on many NLP tasks.
SADR	Severe adverse drug reaction—a particularly harmful or serious adverse drug reaction; often used in the context of signals or reports indicating serious drug-related harms.
SciBERT	A BERT-based model trained on a large corpus of scientific publications (Semantic Scholar) to better handle scientific terminology.
SciFive	A biomedical domain adaptation of the T5 transformer model, pretrained and fine-tuned for various scientific and biomedical NLP tasks.
SE-K	A lightweight model pipeline for surgical note data extraction, which achieves high efficiency and performance (AUROC ~0.94) on registry information extraction tasks.
Sensitivity	True positive rate—the proportion of actual positives correctly identified by a model (recall).
Specificity	True negative rate—the proportion of actual negatives correctly identified by a model.
Transformer	A deep learning model architecture based on self-attention mechanisms, foundational to many modern language models (e.g., BERT, GPT).
Zero-shot learning	A scenario where a model is applied to perform a task without any direct prior examples or training specifically for that task (learning on the fly).

**Table 2:** Human-facing conversational AI or decision support system. This table summarizes studies that evaluated or implemented LLM-based systems designed to support clinicians or researchers through conversational interfaces or integrated decision support tools within clinical workflows.

Ref	Study Objective	LLM Approach	Model Performance	Clinical Impact/Advancement
[16]	Evaluate the capabilities of four advanced LLMs in medication review and reconciliation.	• ChatGPT-3.5 • Gemini Pro • Claude-Instant • LLaMA-2-13B	• Comparative accuracy; Claude/Gemini generally higher • Frequent DDI/genomics errors across models.	• Potential to assist clinical pharmacists • Patients’ safety may be at risk without expert oversight
[17]	Present Clinical nSights platform for real-world oncology analysis using deidentified and harmonized EHR data.	• BERT augmented curation • nCognito de-identification • Graph/distribution harmonization	• Effective phenotype extraction (e.g., tumor markers, irAEs) • Supports Longitudinal data-analysis	• Rapid cohort building and longitudinal dashboards • Enables real-world evidence generation

**Table 3:** Immune-related ADEs. This table summarizes recent studies that applied LLMs to identify or manage immune checkpoint inhibitor (ICI)-related adverse events using clinical text data.

Ref	Study Objective	Clinical Data Source and Size	LLM Approach	Metric and Performance	Clinical Impact/Advancements
[18]	Detect ICI-induced irAEs (MEPS)	Mayo Clinic EHR notes, 9290 patients	• SciBERT fine-tuned (drug-to-phenotype)	Micro-F1 = 0.84 for patient level MEPS identification	• Detected toxicities missed by ICD; • Supports real-world safety
[19]	Detect ICI-induced irAEs	VUMC (100 pts/26,432 notes), UCSF (70 pts/487 notes), Roche (272 pts/272 notes)	• GPT-4o zero-shot (irAE-GPT)	Micro-F1: 0.66 (UCSF), 0.62 (Roche), 0.56 (VUMC) at patient level	• Reduces manual review • Strengthens cross-site safety monitoring
[20]	Detect CART-related AEs	UCSF deidentified clinical data warehouse: 4183 notes/253 patients	• GPT-4 zero-shot (Azure API)	Accuracy = 64.0% (10% sample) at note level	• Automates extraction • Reduces chart review burden for complex CAR-T AEs
[21]	Detect ICI-induced irAEs (adrenal insufficiency, colitis, hypophysitis; ACH).	Mayo Clinic EHR unstructured notes, ~9000 patients	• SciBERT fine-tuned (Augmented Curation)	Micro-F1 = 0.85 at the patient level	• Manual review time reduced from ~9 weeks to ~10 min • Accelerates safety assessments
[22]	Manage ICI-induced IR colitis	Southern Denmark melanoma CPI cohort, 85 patients	• AI language model	BIOx required in 45% of cases	• Reduces prolonged GC exposure • Expedites BIOx initiation
[23]	Detect ICI-induced myocarditis	Progress notes for 1874 ICI admissions (22 myocarditis cases)	• Mistral-7B OpenOrca with RAG (zero-shot)	Sensitivity = 95.5%; Specificity = 95.4% at patient level	• One-day screening enabling early intervention with accuracy
[24]	Detect ICI-induced irAEs	MGH EHR, progress/discharge notes, 7555 admissions (3521 patients)	• Mistral OpenOrca with RAG	Sensitivity = 94.7%; Specificity = 93.7% at patient level	• Chart-review time cut from 15 min to 9.5 s

**Table 4:** Other cancer-related (non-immune) ADEs. This table contains a summary of studies that applied LLMs to detect non-ICI-related ADEs, such as chemotherapy-induced adverse events, using various clinical note types across inpatient and outpatient oncology settings.

Ref	Study Objective	Clinical Data Source and Size	LLM Approach	Metric and Performance	Clinical Impact/ Advancements
[25]	Detect esophagitis presence and severity	Brigham/Dana-Farber EHR—Gold: 1524 notes/124 pts; Silver: 2420 notes/1832 pts; Validation: 345 notes/75 pts	• PubMedBERT fine-tuned (gold + silver)	Macro-F1: 0.92 (any vs. none), 0.82 (grade 2+ vs. ≤1), 0.74 (none vs. grade 1 vs. grade 2–3)	• Detected esophagitis in 100% of test cases vs. 33% by ICD-10, • Enabled enhanced AE detection
[26]	Detect chemotherapy-induced ADEs in breast cancer	OICI breast cancer notes: 1928 notes/434 pts	• Japanese BERT fine-tuned; domain adaptation (800 docs)	Micro-F1 = 0.84 on OICI test set	• Uncovered previously unreported ADEs; • Improved safety surveillance
[27]	Signal detection of chemotherapy-associated AEs	Univ. of Tokyo EMRs: 44,502 pts; 175,624 DPC records; 4.86 M progress, 3.61 M nursing, 122 K discharge notes; median follow-up 1874 days	• MedNERN (BERT fine-tuned on Japanese clinical text)	HRs: 1.63 (phlebitis), 3.85 (oral mucositis), 4.71 (thrombocytopenia), 3.34 (alopecia), all *p* < 0.001	• Reflects known AE frequencies • Enables automated postmarketing surveillance
[28]	Detect taxane-induced peripheral neuropathy	Kyoto Univ Hospital EHR: 215 pts; 159,736 physician, 40,385 nursing, 23,073 discharge, 5663 radiology, 1147 radioisotope, 29,148 pharmacist notes	• MedNERCRJA BERT NER + Levenshtein normalization	Detection rate = 60.7% at 30 days (manual = 74.0%); Pearson r = 0.87	• Near-real-time symptom tracking • Reduces monitoring costs
[29]	Detect chemotherapy-induced AEs in outpatient settings	1008 annotated assessment notes; applied to 2193 ARAT-related clinical notes	• MedNERCRJA (BERT-based NER)	Macro-F1 = 0.72 (exact match) on assessment notes	• Supports pharmacist-led outpatient AE monitoring

**Table 5:** Generalizability of LLMs and low-resource modeling. This table contains a summary of studies that evaluated the generalizability of large language models for adverse event detection across diverse datasets or low-resource settings, including cross-institutional applications and multi-domain benchmark evaluations.

Ref	Study Objective	Clinical Data Source and Size	LLM Approach	Metric and Performance	Clinical Impact/ Advancements
[30]	Benchmark multi-domain ADE extraction	n2c2: 505/66,810; MADE: 1089/61,563; PHEE: 4827/4844; PsyTAR: 3147/3169; CADEC: 1250/7593; CADECv2: 3548/22,389	• GPT-4 in-context (5-shot)	Micro-F1: 92.2% (drug), 51.8% (ADE)—on n2c2; MADE: 91.8% (drug), 68.7% (ADE)	• Enables unified active ADE surveillance across diverse text types
[31]	Extract surgical registry variables from EHR	Stanford Health EHR: 2137 patients/5432 notes	• Majority-vote ensemble: SE-K, SE-E, fine-tuned BioClinicalBERT	AUROC = 0.94 ± 0.04 on 2021 preoperative notes	• Accelerates registry creation • Enables faster surgical outcomes research
[32]	Detect ICI-related ADEs across institutions	ICIOSU: 47 pts/1394 notes; n2c2: 505 notes	• ClinicalBERT fine-tuned (cross-dataset)	Micro-F1 = 0.78 (ICI → n2c2); F1 = 0.74 (n2c2 → ICI)	• Enables generalizable ADE detection across health systems

**Table 6:** Improving pharmacovigilance with high-performance models and advanced methods. This table summarizes studies that utilized high-performing language models and advanced modeling techniques to enhance adverse drug event detection, relation extraction, normalization, and pharmacovigilance workflows across diverse clinical datasets.

Ref	Study Objective	Clinical Data Source and Size	LLM Approach	Metric and Performance	Clinical Impact/ Advancements
[33]	Classify allergy-related symptoms	Songklanagarind Hospital EHR: 79,912 pharmacist-reviewed records	• Ensemble (NBSVM, ULMFiT, XLM-RoBERTa, AllergyRoBERTa)	Exact Match Ratio = 95.33%; Micro-F1 = 98.88%	• Accelerates allergy coding • Reduces pharmacist workload
[34]	Extract dietary supplement AEs and indications	UMN CDR: 7000 sentences (NER); 3000 sentences (RE)	• BERT large cased + CRF (NER) • attention-based BiLSTM (RE)	NER Micro-F1 = 0.86; RE Micro-F1 = 0.89	• Enables dietary supplement safety surveillance using clinical notes
[35]	Extract neuro-AEs, problems, treatments, tests	MTSamples: 163 summaries; VAERS: 91 reports	• GPT-4 with task-specific prompt framework + 5-shot	Relaxed Micro-F1 = 0.861 (MTSamples), 0.736 (VAERS)	• Reduces annotation effort • Enables rapid ADE extraction
[36]	Identify causally related drug–AE pairs	Annotated 5088 segments; Singapore discharge: 1647; MIMIC-III: 4418	• BioMELECTRA-Large fine-tuned + BiLSTM	Micro-F1 = 0.74 (vs. 0.64 traditional)	• Automates ADE causality identification. • Reduces manual review burden
[37]	Normalize medications and ADEs to vocabularies	1.5M EHR notes; MADE: 1089 notes; NCBI: 793 abstracts; CDR: 1500 abstracts	• EhrBERT1M (BioBERT fine-tuned on 1M EHRs)	Micro-F1 = 68.82% (MADE); Macro-F1 = 90.35% (NCBI), 93.82% (CDR)	• Enables large-scale pharmacovigilance and concept normalization
[38]	Extract ADE relations	n2c2-2018: 505 discharge summaries (MIMIC-III)	• BERT fine-tuned	Precision = 0.93; Recall = 0.96; Micro-F1 = 0.94	• Enables real-time ADE surveillance • Reduces clinical risk
[39]	Detect ADR mentions	Austin Health EMR: 861 discharge summaries (311 ADRs)	• MeDeBERTa (DeBERTa pretrained on 1.1M docs, fine-tuned NER)	ROC-AUC = 0.955 (95% CI: 0.933–0.978)	• Bypasses ICD-10 coding • Increases ADR reporting efficiency
[40]	Detect serious AEs from IBD immunosuppressants	UCSF EHR: 928 pts/928 notes (703 SAEs)	• HUCSF-BERT fine-tuned hierarchical model	Macro-F1 = 0.68	• Enables automated drug safety surveillance
[41]	Extract ADE and reason relations	MADE: 21 pts/1092 notes; n2c2 (MIMIC-III): 505 summaries	• BERT fine-tuned + edge sampling	Micro-F1 improvement: +6.4% (MADE), +6.7% (n2c2)	• Improves ADE surveillance accuracy • Enhances postmarketing safety
[42]	Extract bleeding-related entities	Northwestern U discharge summaries: 1079 notes	• BERT variants, LM-LSTM-CRF joint LM	Token-level Macro-F1 = 0.75; Sentence-level = 0.84; Note-level = 0.96	• Supports anticoagulant prescribing by automating bleeding event extraction
[43]	Predict bleeding ADR causality (Naranjo)	UMass EHR: 991 pts/1385 discharge summaries	• ClinicalBERT + logistic regression	Macro-F1 = 0.50; Weighted F1 = 0.63	• Reduces manual chart review in causality assessment
[44]	Classify penicillin ADR risk labels	EHR: 12,232 pts; Synthetic: 69 labels; Test: 1000 cases	• BERT with transfer learning	AUC = 0.995 (high vs. low risk); 0.984 (allergy vs. intolerance)	• Enables automation in penicillin allergy de-labeling workflows
[45]	Identify ADR-related entities	24,890 ADR reports (147,451 annotated entities)	• BBCRadical (BERT + BiLSTM-CRF with radical embeddings)	Micro-F1 = 96.2%; Precision = 96.4%; Recall = 96.0%	• Enhances ADR report quality and postmarketing evaluation
[46]	Detect ADEs using scalable model	n2c2: 505 notes; VUMC: 112 pts/1541 notes	• ClinicalLongformer (split-based 10-chunk)	Macro-F1 = 0.868 (n2c2); F1 = 0.786 (VUMC)	• Enables scalable ADE surveillance from real-world notes

**Table 7:** Pharmacovigilance and drug–drug interaction. This table summarizes studies that applied large language models to identify drug–drug interaction (DDI) signals and support pharmacovigilance efforts using biomedical literature and electronic medical records.

Ref	Study Objective	Clinical Data Source and Size	LLM Approach	Metric and Performance	Clinical Impact/ Advancements
[47]	Identify DDI–severe adverse drug reactions (SADRs) signals	PubMed: 160,321 abstracts (111 DDI–SADR signals); Validation: VUMC (3.18M pts), All of Us (239K pts)	• SciFive PubMed Large fine-tuned	Validated 17 true interactions; 9 novel signals not in DrugBank	• Helps clinicians avoid prescribing PK DDI combinations with unrecognized SADRs
[48]	Identify DDIs increasing isoniazid hepatotoxicity risk	Changsha EMR: 3209 isoniazid pts/128 injury cases; 25,130 ADR records	• Chinese BERT + logistic regression	20 signal drugs identified; strongest OR = 2.69 for cefepime	• Enhances pharmacovigilance by identifying DDI signals for isoniazid safety monitoring

**Table 8:** Decision support and treatment modification. This table contains a summary of studies that used large language models to extract reasons for medication discontinuation or to provide pharmacogenomic-guided treatment recommendations, supporting personalized therapy decisions and improved clinical management.

Ref	Study Objective	Clinical Data Source and Size	LLM Approach	Metric and Performance	Clinical Impact/ Advancements
[49]	Extract semaglutide discontinuation reasons	OMNY Health EHR: 90,955 patients/1.22 M sentences	• Transformer QA model fine-tuned + classification model	Accuracy = 91.4% for reason classification	• Automates discontinuation reason extraction • Enhancing pharmacovigilance
[50]	Extract biologic discontinuation/switching reasons (IBD)	OMNY Health EHRs (4 systems, 2017–2023), 10.6 M notes	• RoBERTa QA → Gemini Pro 1.5 fine-tuned	Accuracy = 94.5% for 7 discontinuation reasons	• Unlocks unstructured EHR insights to inform IBD biologic therapy optimization
[51]	Extract PGx-guided clopidogrel dosing insights	100K patients with paired EHR and exome data	• GPT-4 prompt-based dosage extraction	Pharmacogenomic testing could cut ADEs by 38%	• Enables population-scale PGx decision support; • Could prevent 1 thrombosis per ~30 patients

**Table 9:** Articles or editorials and viewpoints. This table contains a summary of viewpoint and editorial articles discussing the role of AI and large language models in adverse drug event detection, pharmacovigilance, and regulatory systems, highlighting key opportunities, challenges, and implications for clinical practice and policy.

Ref	Main Theme	Opportunities Highlighted	Challenges Discussed	Policy/Practice Implication
[52]	AI-enabled real-world data for oncology pharmacovigilance	Use of NLP for adverse event detection from EHRs; Three Ps framework (Processing, Pipelines, Patient outcomes)	Data labeling issues, causality attribution challenges, data silos, fairness in AI	• Encourages better data sharing, robust labeling, standardization, and focus on health equity in pharmacovigilance design
[53]	AI in detecting and preventing adverse drug events	Applications of AI/ML and LLMs in ADE detection from EHRs, social media, and predictive drug modeling	Inaccuracy of LLMs, limited real-time validation, privacy issues, biases, and system integration challenges	• Pharmacists should lead AI evaluation, advocate for responsible AI use, and integrate AI knowledge into clinical practice
[54]	LLMs in FDA postmarketing surveillance via the Sentinel System	LLMs for signal detection, probabilistic phenotyping, enhancing narrative data use	Infrastructure needs, hallucinations, inference limitations, equity concerns	• Highlights the need for cautious and equitable LLM deployment in regulatory surveillance systems

**Table 10:** Visible gains versus underlying challenges in LLM-based ADE detection.

Area	Visible Gains	Underlying Challenges
Speed and efficiency	• Large reductions in manual review time • Rapid screening enables earlier intervention	• Accuracy and safety still require clinician oversight
Scalability and text mining	• Cohort building and surveillance over millions of notes • Near-real-time dashboards for real-world data	• Cross-site/domain shift may reduce external performance • Data harmonization and provenance tracking are required
Decision support	• PGx-guided and treatment-modification insights • Structured hand-offs can streamline clinician workflow	• Hallucination/mis-advice risk without guard-rails • High-risk interactions must be escalated, not auto-actioned
Generalizability	• Domain adaptation across hospitals and synthetic augmentation can boost performance	• Bias and under-representation from different hospital sites may affect performance.
Interpretability	• Explains why the case was flagged, so gains trusts, helps to spot mistakes quickly, and act faster.	• Keep explanations faithful and brief—only true drivers, with rule checks and calibrated confidence.
Privacy and data sharing	• Federated training can enable multi-site learning	• Privacy constraints limit centralizing raw notes • Robust auditing is still required under federation
Infrastructure and equity	• Lightweight models enable CPU-friendly deployment	• GPU/compute demands and resource gaps may widen disparities
Regulatory and governance	• Sentinel-style surveillance and audit trails emerging	• Standards for hallucinations/false positives still unsettled • Prospective validation and change-control needed

**Table 11:** Immediate and near-term actions for advancing LLM-based ADE detection.

Priority	Concrete Action	Rationale/Example Refs
Immediate	Release open benchmark datasets with site-level (held-out hospital) splits—train on hospital, test on a different, unseen hospital (not used for training) to measure true cross-site generalizability.	Improves cross-site generalizability; MADE, n2c2; Guan 2020 et al. [41]
	Capture clinician feedback via “accept/override” dashboards	Turns false alerts into training data; Sridharan et al. [16]; Koon et al. [36]
	Deploy lightweight CPU-friendly models (e.g., SE-K)	Enables adoption without GPUs; Tavabi et al. [31]
	Embed real-time bias audits (age, sex, race dashboards)	Reveals equity gaps early; Gallifant et al. [52]
Near-term	Federated or differential-privacy fine-tuning across health systems	Robustness without raw data sharing; early EHR pilots
	Add causal explanation layers (counterfactual reasoning)	From heat-maps to actionable justifications, Rawat et al. [43]
	Run prospective, multi-center impact studies with FDA-style audit trails	Regulatory-grade evidence; Matheny et al. [54]
	Extend to multimodal ADE detection (text + labs ± imaging)	Captures false negatives of text-only models; Staneková et al. [44]

## Data Availability

No new data were created in this study. All information discussed is derived from the literature cited in the manuscript; therefore, data sharing is not applicable.
